# Supplementary material for: Modeling glioblastoma heterogeneity as a dynamic network of cell states
Source: Mol Syst Biol. 2021 Sep 16;17(9):e10105. doi: 10.15252/msb.202010105 (PMC8444284; doi:10.15252/msb.202010105)
Supplement: Supplementary file 6 — Source Data for Figure 5 [file MSB-17-e10105-s004.zip › Figure5A_sourcedata/GSEA_3071/nosigdb_stateII.GseaPreranked.1622795151171/RICHARDS_NOWAKOWSKI_SCIENCE_2017_IN_CTX_CGE2_UPREG.html]

Details for gene set RICHARDS\_NOWAKOWSKI\_SCIENCE\_2017\_IN\_CTX\_CGE2\_UPREG[GSEA]

|  || Dataset | state23071 |
| Phenotype | NoPhenotypeAvailable |
| Upregulated in class | na\_neg |
| GeneSet | RICHARDS\_NOWAKOWSKI\_SCIENCE\_2017\_IN\_CTX\_CGE2\_UPREG |
| Enrichment Score (ES) | -0.5173623 |
| Normalized Enrichment Score (NES) | -2.937258 |
| Nominal p-value | 0.0 |
| FDR q-value | 0.0 |
| FWER p-Value | 0.0 |
Table: GSEA Results Summary

  

Fig 1: Enrichment plot: RICHARDS\_NOWAKOWSKI\_SCIENCE\_2017\_IN\_CTX\_CGE2\_UPREG      
 Profile of the Running ES Score & Positions of GeneSet Members on the Rank Ordered List

  

| PROBE | GENE SYMBOL | GENE\_TITLE | RANK IN GENE LIST | RANK METRIC SCORE | RUNNING ES | CORE ENRICHMENT || 1 | ATAD5 |  |  | 18 | 1.469 | 0.0150 | No |
| 2 | SSRP1 |  |  | 478 | 0.638 | -0.0918 | No |
| 3 | CALB2 |  |  | 485 | 0.636 | -0.0849 | No |
| 4 | NOP58 |  |  | 538 | 0.609 | -0.0898 | No |
| 5 | SMC1A |  |  | 618 | 0.570 | -0.1021 | No |
| 6 | NETO2 |  |  | 740 | 0.519 | -0.1256 | No |
| 7 | RIMS1 |  |  | 776 | 0.506 | -0.1277 | No |
| 8 | SNRPA1 |  |  | 954 | 0.458 | -0.1661 | No |
| 9 | CRIM1 |  |  | 959 | 0.456 | -0.1610 | No |
| 10 | KPNA3 |  |  | 1046 | 0.429 | -0.1769 | No |
| 11 | TOMM5 |  |  | 1049 | 0.428 | -0.1717 | No |
| 12 | EIF3J |  |  | 1159 | 0.405 | -0.1937 | No |
| 13 | NR2F2 |  |  | 1264 | 0.384 | -0.2147 | No |
| 14 | ITGB1BP1 |  |  | 1382 | 0.360 | -0.2393 | No |
| 15 | ANKRD36C |  |  | 1404 | 0.357 | -0.2399 | No |
| 16 | CCT3 |  |  | 1446 | 0.347 | -0.2456 | No |
| 17 | EIF5A |  |  | 1461 | 0.346 | -0.2445 | No |
| 18 | TRIO |  |  | 1493 | 0.342 | -0.2478 | No |
| 19 | ANKRD36B |  |  | 1495 | 0.341 | -0.2435 | No |
| 20 | WBP11 |  |  | 1549 | 0.331 | -0.2524 | No |
| 21 | PSMA3 |  |  | 1692 | 0.312 | -0.2839 | No |
| 22 | DRG1 |  |  | 1693 | 0.312 | -0.2798 | No |
| 23 | VCAN |  |  | 1793 | 0.299 | -0.3006 | No |
| 24 | HSPA9 |  |  | 1884 | 0.287 | -0.3194 | No |
| 25 | ANKRD36 |  |  | 1953 | 0.279 | -0.3328 | No |
| 26 | TNPO3 |  |  | 1989 | 0.274 | -0.3380 | No |
| 27 | KITLG |  |  | 2019 | 0.270 | -0.3417 | No |
| 28 | NR2F1 |  |  | 2071 | 0.265 | -0.3509 | No |
| 29 | CAMK2N1 |  |  | 2111 | 0.262 | -0.3573 | No |
| 30 | EIF2AK2 |  |  | 2148 | 0.257 | -0.3629 | No |
| 31 | KALRN |  |  | 2182 | 0.254 | -0.3678 | No |
| 32 | CBX1 |  |  | 2210 | 0.251 | -0.3713 | No |
| 33 | BAG5 |  |  | 2241 | -0.253 | -0.3754 | No |
| 34 | SYT14 |  |  | 2296 | -0.258 | -0.3856 | No |
| 35 | PPP2CB |  |  | 2394 | -0.270 | -0.4063 | No |
| 36 | LUC7L |  |  | 2430 | -0.275 | -0.4115 | No |
| 37 | DCUN1D1 |  |  | 2667 | -0.311 | -0.4666 | No |
| 38 | EP300 |  |  | 2670 | -0.312 | -0.4630 | No |
| 39 | IGF1R |  |  | 2700 | -0.316 | -0.4661 | No |
| 40 | SOGA1 |  |  | 2731 | -0.322 | -0.4693 | No |
| 41 | BOD1L1 |  |  | 2734 | -0.323 | -0.4655 | No |
| 42 | GAS2L3 |  |  | 2755 | -0.327 | -0.4662 | No |
| 43 | PRKCA |  |  | 2757 | -0.327 | -0.4621 | No |
| 44 | ZNF302 |  |  | 2809 | -0.337 | -0.4704 | No |
| 45 | PAK3 |  |  | 2839 | -0.343 | -0.4732 | No |
| 46 | GNPTAB |  |  | 2862 | -0.346 | -0.4741 | No |
| 47 | ARID1B |  |  | 2926 | -0.360 | -0.4852 | No |
| 48 | ELOVL6 |  |  | 2958 | -0.366 | -0.4881 | No |
| 49 | TMOD2 |  |  | 2985 | -0.370 | -0.4897 | No |
| 50 | MIAT |  |  | 2996 | -0.371 | -0.4873 | No |
| 51 | CIR1 |  |  | 3007 | -0.375 | -0.4848 | No |
| 52 | KANSL1 |  |  | 3045 | -0.384 | -0.4890 | No |
| 53 | AKAP9 |  |  | 3066 | -0.389 | -0.4889 | No |
| 54 | AP1S2 |  |  | 3169 | -0.419 | -0.5089 | No |
| 55 | MIDN |  |  | 3175 | -0.421 | -0.5046 | No |
| 56 | FAM110B |  |  | 3191 | -0.426 | -0.5027 | No |
| 57 | CHMP1B |  |  | 3207 | -0.430 | -0.5008 | No |
| 58 | RTN1 |  |  | 3232 | -0.436 | -0.5010 | No |
| 59 | NPAS3 |  |  | 3233 | -0.437 | -0.4952 | No |
| 60 | TBC1D23 |  |  | 3245 | -0.440 | -0.4921 | No |
| 61 | PDZRN3 |  |  | 3276 | -0.449 | -0.4937 | No |
| 62 | CGGBP1 |  |  | 3280 | -0.451 | -0.4885 | No |
| 63 | BRD3 |  |  | 3346 | -0.474 | -0.4985 | No |
| 64 | APC |  |  | 3422 | -0.510 | -0.5106 | Yes |
| 65 | UBXN7 |  |  | 3430 | -0.511 | -0.5056 | Yes |
| 66 | ARX |  |  | 3443 | -0.516 | -0.5017 | Yes |
| 67 | PAK2 |  |  | 3446 | -0.518 | -0.4954 | Yes |
| 68 | RAB11B |  |  | 3451 | -0.521 | -0.4895 | Yes |
| 69 | QKI |  |  | 3467 | -0.526 | -0.4863 | Yes |
| 70 | UQCR11 |  |  | 3469 | -0.527 | -0.4795 | Yes |
| 71 | ASAP1 |  |  | 3484 | -0.534 | -0.4759 | Yes |
| 72 | WDR83OS |  |  | 3533 | -0.562 | -0.4805 | Yes |
| 73 | INTU |  |  | 3540 | -0.565 | -0.4745 | Yes |
| 74 | GNG2 |  |  | 3543 | -0.567 | -0.4675 | Yes |
| 75 | WWP1 |  |  | 3581 | -0.592 | -0.4690 | Yes |
| 76 | CD24 |  |  | 3624 | -0.620 | -0.4713 | Yes |
| 77 | GNAQ |  |  | 3660 | -0.653 | -0.4714 | Yes |
| 78 | ASAH1 |  |  | 3666 | -0.655 | -0.4640 | Yes |
| 79 | NDUFB4 |  |  | 3678 | -0.668 | -0.4579 | Yes |
| 80 | GAD1 |  |  | 3704 | -0.690 | -0.4550 | Yes |
| 81 | PCDH9 |  |  | 3707 | -0.691 | -0.4463 | Yes |
| 82 | RGS12 |  |  | 3714 | -0.697 | -0.4386 | Yes |
| 83 | CXCR4 |  |  | 3733 | -0.710 | -0.4337 | Yes |
| 84 | DLX6 |  |  | 3735 | -0.711 | -0.4245 | Yes |
| 85 | NRXN3 |  |  | 3738 | -0.714 | -0.4155 | Yes |
| 86 | ZNF704 |  |  | 3750 | -0.729 | -0.4086 | Yes |
| 87 | SOX4 |  |  | 3797 | -0.786 | -0.4097 | Yes |
| 88 | MAGI1 |  |  | 3827 | -0.815 | -0.4062 | Yes |
| 89 | PAIP2 |  |  | 3830 | -0.821 | -0.3958 | Yes |
| 90 | PIAS2 |  |  | 3848 | -0.840 | -0.3890 | Yes |
| 91 | CCDC88A |  |  | 3854 | -0.848 | -0.3790 | Yes |
| 92 | SLAIN1 |  |  | 3862 | -0.855 | -0.3694 | Yes |
| 93 | SLC5A3 |  |  | 3867 | -0.876 | -0.3588 | Yes |
| 94 | ZEB2 |  |  | 3876 | -0.894 | -0.3489 | Yes |
| 95 | CADPS |  |  | 3877 | -0.894 | -0.3371 | Yes |
| 96 | CHD7 |  |  | 3880 | -0.904 | -0.3256 | Yes |
| 97 | ROBO1 |  |  | 3920 | -1.005 | -0.3220 | Yes |
| 98 | TMTC4 |  |  | 3933 | -1.023 | -0.3115 | Yes |
| 99 | RAB3IP |  |  | 3940 | -1.037 | -0.2992 | Yes |
| 100 | DCLK2 |  |  | 3945 | -1.043 | -0.2864 | Yes |
| 101 | SDK2 |  |  | 3948 | -1.048 | -0.2730 | Yes |
| 102 | RNF180 |  |  | 3949 | -1.052 | -0.2590 | Yes |
| 103 | PDP1 |  |  | 3954 | -1.075 | -0.2458 | Yes |
| 104 | NCAM1 |  |  | 3966 | -1.106 | -0.2339 | Yes |
| 105 | TCF4 |  |  | 3984 | -1.173 | -0.2226 | Yes |
| 106 | CDH2 |  |  | 3998 | -1.233 | -0.2095 | Yes |
| 107 | NFIB |  |  | 4007 | -1.310 | -0.1941 | Yes |
| 108 | SLCO5A1 |  |  | 4015 | -1.362 | -0.1778 | Yes |
| 109 | CHL1 |  |  | 4019 | -1.379 | -0.1603 | Yes |
| 110 | PROX1 |  |  | 4034 | -1.513 | -0.1437 | Yes |
| 111 | DLX1 |  |  | 4041 | -1.558 | -0.1245 | Yes |
| 112 | CADM1 |  |  | 4042 | -1.568 | -0.1037 | Yes |
| 113 | DLX2 |  |  | 4055 | -1.704 | -0.0841 | Yes |
| 114 | LRRN3 |  |  | 4064 | -1.810 | -0.0621 | Yes |
| 115 | DLX5 |  |  | 4090 | -2.619 | -0.0337 | Yes |
| 116 | DCX |  |  | 4091 | -2.671 | 0.0018 | Yes |
Table: GSEA details [plain text format]

  

Fig 2: RICHARDS\_NOWAKOWSKI\_SCIENCE\_2017\_IN\_CTX\_CGE2\_UPREG: Random ES distribution      
 Gene set null distribution of ES for **RICHARDS\_NOWAKOWSKI\_SCIENCE\_2017\_IN\_CTX\_CGE2\_UPREG**
